# Piezoelectric Current Generator Based on Bismuth Ferrite Nanoparticles

**DOI:** 10.3390/s20236736

**Published:** 2020-11-25

**Authors:** Farid Orudzhev, Shikhgasan Ramazanov, Dinara Sobola, Nariman Alikhanov, Vladimír Holcman, Lubomír Škvarenina, Pavel Kaspar, Gamzat Gadjilov

**Affiliations:** 1 Department of Inorganic Chemistry and Chemical Ecology, Dagestan State University, Makhachkala, st. M. Gadjieva 43-a, 367015 Dagestan Republic, Russia; farid-stkha@mail.ru (F.O.); ramazanv@mail.ru (S.R.); sobola@feec.vutbr.cz (D.S.); alikhanov.nariman@gmail.com (N.A.); gadjilov@bk.ru (G.G.); 2 Department of Physics, Faculty of Electrical Engineering and Communication, Brno University of Technology, Technicka 2848/8, 616 00 Brno, Czech Republic; xskvar01@stud.feec.vutbr.cz (L.Š.); kasparp@feec.vutbr.cz (P.K.); 3 Central European Institute of Technology BUT, Purkyňova 123, 612 00 Brno, Czech Republic

**Keywords:** BiFeO_3_, piezoelectric generator, composite material, nanoparticles

## Abstract

Bismuth ferrite nanoparticles with an average particle diameter of 45 nm and spatial symmetry R3c were obtained by combustion of organic nitrate precursors. BiFeO_3_-silicone nanocomposites with various concentrations of nanoparticles were obtained by mixing with a solution of *M10* silicone. Models of piezoelectric generators were made by applying nanocomposites on a glass substrate and using aluminum foil as contacts. The thickness of the layers was about 230 μm. There was a proportional relationship between the different concentrations of nanoparticles and the detected potential. The output voltages were 0.028, 0.055, and 0.17 V with mass loads of 10, 30, and 50 mass%, respectively.

## 1. Introduction

Piezo nanogenerators (PNGs) are promising sources of electricity that can power small electronics. Piezoelectric materials consist of both inorganic and organic components [1]. Since inorganic piezoelectric materials have better piezoelectric properties, they are the most widely studied. In turn, inorganic piezoelectric materials are classified into piezo crystals and piezo ceramics [2,3]. Research has mostly focused on piezoelectric materials, such as piezoelectric zinc oxide (ZnO) crystals [4,5,6,7,8] and piezoelectric lead zirconate-titanate (PZT) ceramics [9,10,11]. The good piezoelectric characteristics of ZnO in the form of nanowires are a consequence of its unique noncentrosymmetric structure. The method of obtaining them by growing on a substrate, however, is a long procedure that requires expensive equipment. In addition, it was reported that ZnO nanowires are characterized by a number of fundamental limitations associated with their semiconducting nature, for example, high leakage currents. They shield the piezoelectric potential formed in the material and require a Schottky barrier or p-n junction to achieve good energy-harvesting characteristics [4,12,13]. PZT is a widely used ceramic piezoelectric and has a high piezoelectric coefficient, excellent dielectric properties, [11,14,15] and ideal parameters for converting mechanical energy into electricity. However, due to the increased fragility of the thin film, PZT is not applicable for flexible and stretchable systems. The maximum safe mechanical stress for PZT is 0.2%, which indicates structural deformation even at low tension [16]. At the same time, one should not forget about the high toxicity of lead compounds.

As a result, the issue of creating a nontoxic piezoelectric material with high plasticity and low leakage currents remains relevant. One of the potential candidates for widespread use is lead-free perovskite bismuth ferrite (BiFeO_3_). Practical interest in this material has manifested due to its simultaneous ferroelectric and antiferromagnetic states, with extremely high ordering temperatures (Curie temperature *T*_C_ = 830 °C and Néel temperature *T*_N_ = 370 °C) [17]. The presence of ferroelectric ordering leads to the appearance of spontaneous polarization. From the empirical relationship ***P***_S_ = (258 ± 9) Δ*z* μC/cm^2^, established by Abrahams et al. [18], it is assumed that for bulk BiFeO_3_ the ***P***_S_ values can be 98–108 μC/cm^2^. In practice, however, significantly lower values were observed. Although such values were not achieved for bulk ceramic materials, it was theoretically predicted that the ferroelectric properties of nanosized BiFeO_3_ would increase with decreasing particle size [19]. At the same time, measurements on thin films of high quality [20], single crystals [21] and ceramic polycrystals [22] showed that ***P***_S_ is about 60–100 μC/cm^2^ along the polar axis, which is consistent with Abrahams et al. This difference in ***P***_S_ values is explained by the presence of secondary phases, defects, volatilization of bismuth atoms at high temperatures, and electron hopping between Fe ions, which lead to high values of leakage currents [23]. Thus, for practical applications, it is of interest to synthesize phase-pure and nanosized BiFeO_3_ with particle sizes less than 62 nm.

In this work, a promising composite material based on BiFeO_3_ nanoparticles was obtained; the PNG model was assembled on this basis, and the electrical properties of the device were investigated.

## 2. Experimental Technique

### 2.1. Synthesis

The BiFeO_3_ nanoparticles were synthesized by solution combustion. The synthesis process is as follows. The starting reagents Bi(NO_3_)_3_∙5H_2_O and Fe(NO_3_)_3_∙9H_2_O (molar ratio of Bi^3+^ and Fe^3+^ 1:1, with purity > 98) were dissolved in distilled water. Glycine (C_2_H_5_NO_2_, purity > 98) was added to the mixture on the basis of calculations regarding the concept of fuel chemistry [24]. The synthesis process consists of three main steps: formation of a combustion mixture, formation of a gel, and then combustion of a gel. The solution was heated at ~300 °C for 1.5 h until dehydration and was stirred with a magnetic stirrer for homogenization. As a result, the formed gel ignited spontaneously with the release of gases and the formation of a powder. The synthesized powder was calcined at a temperature of 600 °C (Furnance: Nabertherm LF-15/14) in air. The desired temperature was reached at a rate of 5 °C per minute and held for 30 min. Details of the synthesis technology are presented in our previous work [25].

### 2.2. Preparation and Measurement Technique

The assembly of the PNG model was carried out as follows:

1. silicone solution (Super Mold M10, Guangzhou, China) was prepared by adding a curing catalyst (where the weight ratio of silicone to curing agent was 10:1);
2. the synthesized BiFeO_3_ nanopowder was dispersed into the mixture at various concentrations of 10, 30, and 50 mass%;
3. the prepared composite was applied to an Al electrode glued by double-sided tape on glass (2 × 2.5 cm) by spin-coating in a laboratory centrifuge (Liston C 2204 Classic, Liston, Zhukov, Russia) for 10 s at 3000 rpm;
4. the samples were cured in a drying oven at 50 °C for 20 min;
5. another aluminum electrode was glued on top of the BiFeO_3_-silicone composite film, and the material was left to cure completely for a day.

X-ray structural studies were carried out using an Empyrean PANalytical X-ray diffractometer (Almelo, The Netherlands) in the radiation of a copper anode with a nickel filter. Data processing was carried out using the HighScore Plus application program and the PDF-2 diffraction database.

The crystallite sizes were calculated using the Debye–Scherer formula on the broadening of reflections in diffraction patterns:

(1) $$d=\frac{k\lambda }{\beta \cdot \cos\theta }$$

where d is the average crystallite size, λ is the wavelength of the radiation λ(CuK_α_) = 0.154051 nm, *β* is the peak width at half maximum, *θ* is the diffraction angle, and k = 0.9.

The morphology of the obtained samples was studied using a scanning electron microscope (SEM) LEO-1450 (Leica Microsystems Wetzlar Gmbh, Wetzlar, Germany). The Raman spectra were studied using a laser 3D Raman scanning confocal microscope (Ntegra Spectra, Moscow, Russia) using a green laser (532 nm) with a spot size of 1 μm and a resolution of 0.5 cm^−1^. The output voltage and current from PNG under manual mechanical action were recorded using a Keithley 2400 calibrator-multimeter.

## 3. Results and Discussion

**Figure 1:**
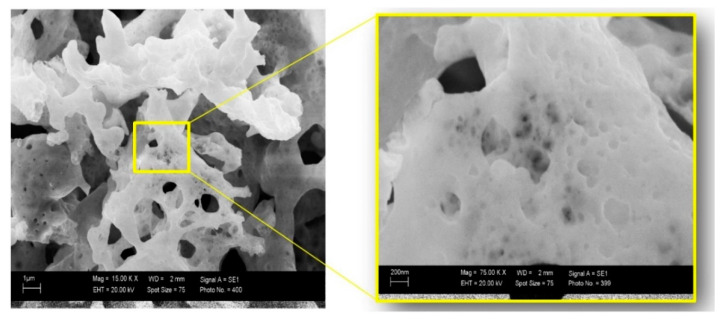
Scanning electron microscope (SEM) image of freshly synthesized bismuth ferrite at different magnification.

As seen with the scanning electron microscope (SEM) (Figure 1), the samples represented the agglomerated nanoparticles in a highly porous structure. The pores were irregular in shape, and their sizes varied in the submicron range. At the same time, cavities were created due to the rapid release of combustion gases [26].

**Figure 2:**
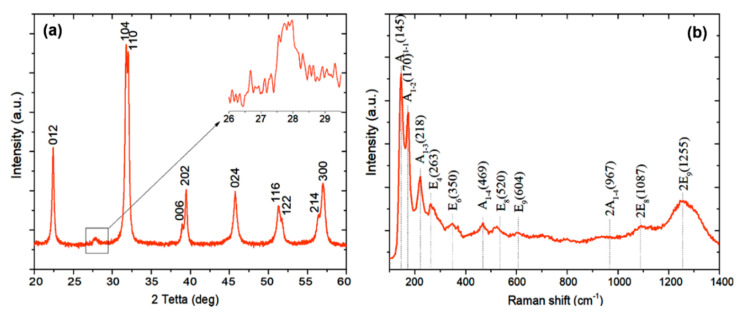
(**a**) X-ray diffraction pattern of freshly synthesized BiFeO_3_; (**b**) Raman spectrum of BiFeO_3_ nanoparticles.

Figure 2a shows a typical X-ray powder diffractogram of bismuth ferrite BiFeO_3_. All marked peaks characterized hexagonal BiFeO_3_ with the spatial symmetry R3c, with a small content of the secondary phase indicated in the inset of Figure 2a.

The average crystallite size of BiFeO_3_ nanopowders determined from the Debye–Scherer equation using (012) and (024) planes was 45 nm. The relatively small peak shown in the inset corresponds to Bi_24_Fe_2_O_39_ located at 2θ = 27.97° (PDF 420201) and Bi_2_Fe_4_O_9_ located at 2θ = 28.2° (PDF 250090).

For a more accurate analysis of the structure of the nanopowder, Raman spectra were obtained (Figure 2b). It is known that the rhombohedral structure of R3c causes 13 active combination modes: 4A_1_ + 9E. The obtained spectrum was characterized by eight modes, four of which belonged to mode A_1_ and four to E. In addition, there were three overtones in the spectrum. The vibrational modes of Bi atoms were present mainly up to 167 cm^−1^. Oxygen atoms had strong vibrations at values above 262 cm^−1^. Fe atoms were mainly involved in modes between 152 and 261 cm^−1^, but also contributed to the appearance of some modes with higher wavenumbers [27,28]. As noted in [29], the mobility of ferroelectric domains is suppressed in the R3c phase due to the interaction of octahedral antiphase boundaries with non-180° domain walls, which makes a significant contribution to the piezoelectric response. According to the structural analysis, the sample had a hexagonal structure with R3m spatial symmetry. This is due to a feature of high-temperature synthesis, where the R3c→R3m transition is observed in BiFeO_3_ [30]. This effect was not associated with a change in the cell parameters. Thus, one can expect an enhancement of the piezoelectric properties of BiFeO_3_ in the R3m phase due to the gradual release of domain walls as the transition proceeds.

**Figure 3:**
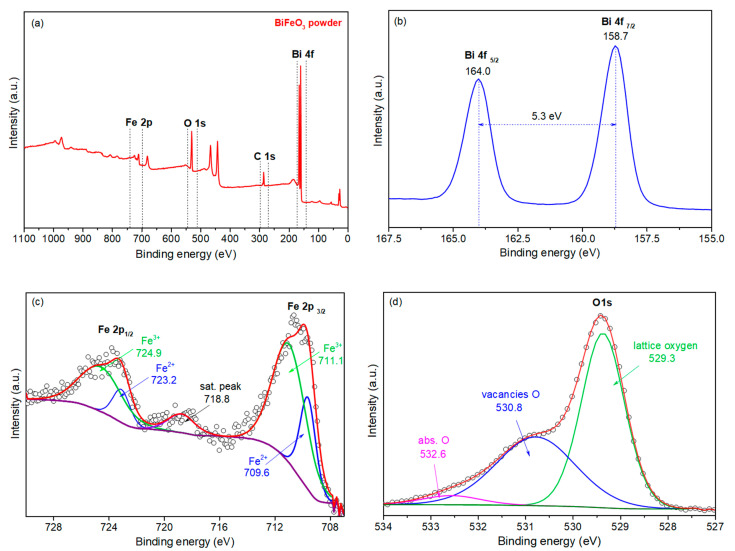
X-ray photoelectron spectroscopy (XPS) spectra of the BiFeO_3_ powder: (**a**) survey spectrum, (**b**) Bi 4f spectrum, (**c**) Fe 2p spectrum, (**d**) O 1s spectrum.

The chemical composition of the powder was studied by X-ray photoelectron spectroscopy (XPS) using an AXIS SupraTM X-ray photoelectron spectrometer (Kratos Analytical Ltd., Manchester, UK). The spectra (Figure 3) were calibrated by C1s peak at 284.8 eV.

High-resolution XPS spectra are shown in Figure 3a. Peaks Bi 4f_7/2_ and Bi 4f_5/2_ were located at ~159.0 eV and ~164.3 eV. In addition, we note that the energy of the spin-orbit splitting of the Bi 4f doublet, which is equal to the energy difference between the Bi 4f_7/2_ and Bi 4f_5/2_ peaks, was 5.3 eV, which agrees with the previously reported experimental and theoretically calculated values. Thus, the valence state of Bi ions can be defined as 3+. The spectra of the core levels of Fe 2p are shown in Figure 3c. The asymmetric nature of the peaks and the presence of satellite peaks indicate the presence of iron in the oxidation states Fe^2+^ and Fe^3+^. Peak deconvolution delineates the overlapping Fe^2+^ and Fe^3+^ peaks. A high-resolution spectrum of the O 1s state was also obtained. A strong peak at 529.7 eV is assigned to the characteristic signal from lattice O. The shoulder, at about 531.5 eV, is assigned to defective O components, such as oxygen vacancies.

The core level peaks of iron, bismuth, and oxygen are highlighted at the survey spectrum (Figure 3a) and were chosen for further processing. The peak of bismuth Bi 4f was characterized with asymmetric duplets of spin-orbit components. The splitting of 5.3 eV and the positions of Bi 4f_7/2_ and Bi 4f_5/2_ at 158.7 eV and 164.0 eV (correspondingly) confirmed the presence of oxidized bismuth in the powder (Figure 3b) and the presence of defect states (Figure 3c) [31,32].

**Figure 4:**
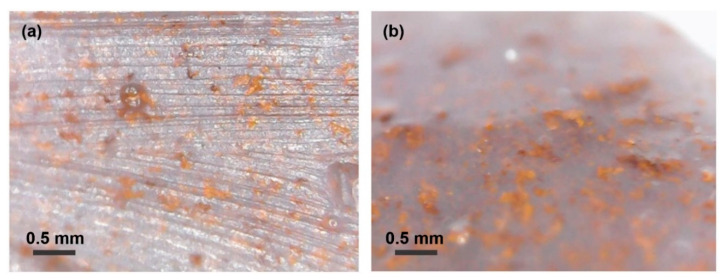
Optical images of the BiFeO_3_-silicone composite at 500× magnification. The figures show the filling by the powder in the volume of silicone: (**a**) sample cross-section, (**b**) sample top view.

Figure 4 shows optical micrographs for the obtained BiFeO_3_-silicone composites with the different mass loading of BiFeO_3_. With this approach of obtaining a composite, it is shown that a uniform distribution of nanoparticles is observed over the entire volume of the silicone matrix.

**Figure 5:**
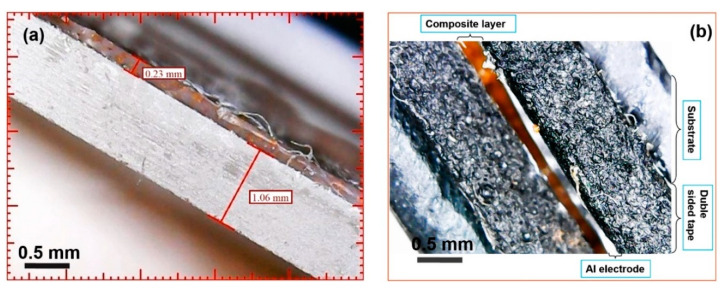
(**a**) Optical micrograph of the BiFeO_3_-silicone composite film at 500× times magnification; (**b**) optical micrograph of the piezo nanogenerator (PNG) device at 500× magnification.

Figure 5a shows an optical micrograph of a cleavage of a BiFeO_3_-silicone composite film obtained by centrifugation at 500× *g* magnification. It can be seen from the photograph that a homogeneous film with a thickness of about 230 μm was formed. Figure 5b shows an optical micrograph of a cleavage of the assembled PNG device.

**Figure 6:**
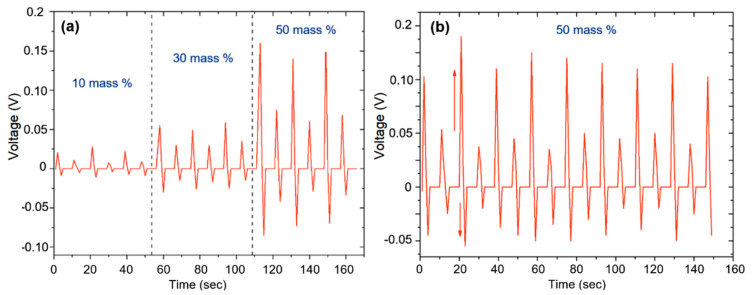
(**a**) Voltage generated in the PNG circuit with different concentrations of BiFeO_3_ loading; (**b**) the lower graph shows the character of the potential change on the composition for a longer time, where the increase in stress during compression and relaxation is seen.

The typical dependencies of the output voltage on time, measured for PNG with different loading concentrations (10, 30, and 50 mass%) with successive compression and relaxation, are shown in Figure 6. The generated output voltages of unpolarized PNG were measured under periodic vertical compression and relaxation.

The output voltages were about 0.028, 0.055, and 0.17 V for PNG loaded with 10, 30, and 50 mass% BiFeO_3_, respectively. The output voltage gradually increased with an increase in the content of BiFeO_3_ nanoparticles in the composite of up to 50 mass%, reaching a maximum output voltage of about 0.17 V. The results show that the PNG model obtained in this work demonstrated a performance comparable to the characteristics of other PNGs [33,34]. The observed difference in peak stress values between the states during compression and relaxation can be associated with the difference in the rate of PNG deformation during compression and relaxation. 

**Figure 7:**
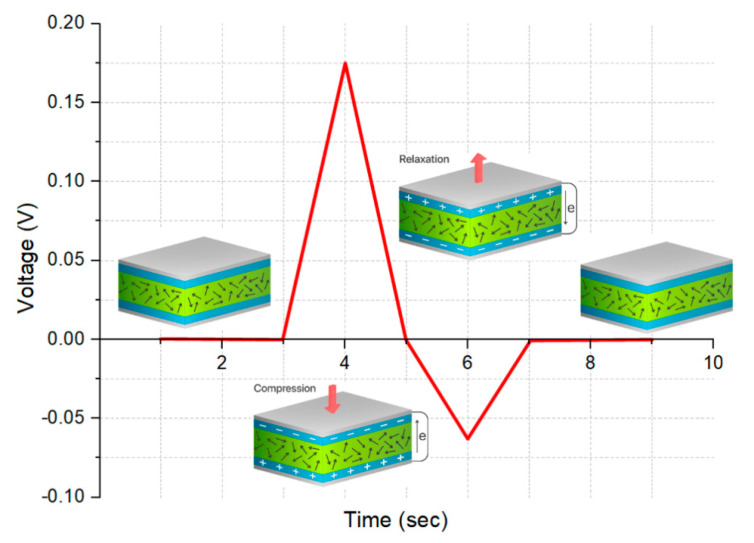
Schematic representation and electrical response of the PNG operating mechanism.

Figure 7 schematically shows the PNG device’s mechanism of operation. It is known that, without polarization, electric dipoles in BiFeO_3_ nanoparticles are directed randomly [35].

Without the application of an external mechanical force, the output voltage is not recorded on the PNG since the device is in a state of equilibrium. Upon vertical compression, however, the polarization of the composite changes due to the compression deformation. This leads to the appearance of a piezoelectric potential between the upper and lower electrodes, and the movement and accumulation of free charges on the electrodes. Circuit voltage of PNG during this process with a BiFeO_3_ nanoparticle concentration of 10 mass% is shown in Figure 7.

The vertical deformation and piezoelectric potential between the two electrodes disappeared at relaxation. The accumulated charges moved in the opposite direction, and a negative electrical signal was generated (Figure 7).

Non-polarized PNG samples produce low voltage values. When PNG is polarized by applying an electric field, the dipoles in BiFeO_3_ nanoparticles will be aligned along the direction of the applied electric field, and so a significant increase in the output voltage will be observed.

## 4. Conclusions

The novelty of this work is in the piezoelectric generator, prepared with bismuth ferrite nanosized powder in a silicon solution. The structure and composition of the powder were analyzed in detail. The active layer is represented by BiFeO_3_-silicone nanocomposite. The active layer thickness was 230 µm. The output voltage depends on the concentration of the powder in the silicon solution. The mechanism of PNG operation is considered. All the results from measurements presented in this paper show that the manufacturing process described yields a suitable piezoelectric material to use for nanogenerators.
